# Correlation between periodontal maintenance compliance and implant survival following fixed prosthodontic restorations

**DOI:** 10.6026/973206300223681

**Published:** 2026-06-30

**Authors:** Gatha Mohanty1, Ramnath Revankar, Vipul Asopa, Shivalika S. Asopa, Garima Rani, P. Arun Kumar, Guljot Singh

**Affiliations:** 1 Department of Periodontics and Oral Implantology, Institute of Dental Science, Siksha 'O' Anusandhan deemed to be University, Bhubaneswar, Odisha, India; 2 Department of Prosthodontics, Ranjeet Deshmukh Dental College, Nagpur, India; 3 Department of Prosthodontics, Pacific Dental College & Research Centre, Udaipur, India; 4 Department of Public Health Dentistry, Maharishi Markandeshwar College of Dental Sciences & Research (MMCDSR), Haryana, India; 5 Department of Prosthodontics and Crown & Bridge, Priyadarshini Dental College and Hospital, Thiruvallur, Chennai, India; 6 Department of Periodontology & Implantology, Daswani Dental College & Research Center, Kota, Rajasthan, India

**Keywords:** Periodontal maintenance, implant survival, fixed prosthodontic restorations, dental implants, supportive periodontal therapy, patient compliance

## Abstract

Periodontal maintenance compliance plays a crucial role in the long-term success of implant-supported fixed prosthodontic restorations. This retrospective cohort study evaluated the correlation between periodontal maintenance compliance and implant survival among 100 patients with fixed implant-supported prostheses. The overall implant survival rate was 93%, with compliant patients demonstrating significantly higher survival rates (96.8%) compared to non-compliant patients (86.8%) (P < 0.05), along with a moderate positive correlation between maintenance compliance and implant survival (r = 0.41, p < 0.01). Logistic regression analysis identified periodontal maintenance compliance as a significant independent predictor of implant survival. The findings suggest that regular supportive periodontal therapy and patient adherence to maintenance programs are essential for improving long-term implant success and minimizing biological complications.

## Background:

Dental implants are a widely accepted treatment option for replacing missing teeth in fixed prosthodontic rehabilitation. Long-term studies have shown high implant survival rates with improved esthetics, function and patient satisfaction [1]. Despite favourable outcomes, implant failure and biological complications remain important clinical concerns. Implant survival is influenced by bone quality, surgical technique, prosthetic loading and oral hygiene practices [2]. Peri-implant tissue health is considered a major factor affecting long-term implant success. Peri-implant tissues are biologically more vulnerable to plaque-induced inflammation than natural periodontal tissues [3]. Patients with fixed implant-supported prostheses often experience difficulty maintaining proper oral hygiene due to limited access for cleaning and inadequate awareness regarding implant care [4]. Poor plaque control may result in peri-implant mucositis and progression to peri-implantitis, ultimately leading to bone loss and implant failure [5]. Supportive periodontal therapy plays an essential role in preserving peri-implant tissue health through professional cleaning, clinical evaluation and reinforcement of oral hygiene practices [6]. Studies have reported that patients who are compliant with periodontal maintenance demonstrate greater periodontal stability and fewer peri-implant complications than non-compliant individuals [7]. However, maintenance compliance is often influenced by factors such as lack of motivation, financial constraints and time limitations [8]. Non-compliant patients frequently show increased plaque accumulation, deeper probing depths and greater marginal bone loss around implants [9]. Furthermore, patients with a history of periodontitis are at greater risk of peri-implant disease when maintenance care is inadequate [10]. Although implant survival and peri-implant diseases have been extensively studied, limited evidence is available regarding the direct correlation between periodontal maintenance compliance and implant survival in patients with fixed prosthodontic restorations. Therefore, it is of interest to evaluate the correlation between periodontal maintenance compliance and implant survival in patients rehabilitated with fixed prosthodontic restorations.

## Methodology:

All data for the present retrospective cohort study were obtained from patient records and entered into Microsoft Excel before being analysed using the Statistical Package for the Social Sciences (SPSS) software (Version 26.0; IBM Corp., Armonk, NY, USA). Sample size estimation was performed using G*Power software (Version 3.1.9.7; Heinrich Heine University Düsseldorf, Germany). Assuming a moderate association between periodontal maintenance compliance and implant survival (effect size = 0.35), with an alpha level of 0.05 and 80% power, the minimum required sample size was calculated to be 78 patients. To enhance the reliability of the results and compensate for incomplete records, 100 patients with fixed implant-supported prosthodontic restorations were included in the analysis. Descriptive statistics were used to summarise the data, with continuous variables expressed as mean and standard deviation and categorical variables presented as frequencies and percentages. Periodontal maintenance compliance was categorised as compliant and non-compliant based on the frequency of follow-up visits. The association between periodontal maintenance compliance and implant survival was assessed using the Chi-square test. Spearman's correlation analysis was performed to evaluate the strength of the relationship between maintenance compliance and implant survival duration. Multivariate logistic regression was used to assess the effect of periodontal maintenance compliance on implant survival, adjusting for potential confounders, including age, gender, smoking status and history of periodontal disease. All statistical tests were two-tailed and a p-value of less than 0.05 was considered statistically significant.

## Results:

A total of 100 patients with fixed implant-supported prosthodontic restorations were included in the present retrospective study. Based on periodontal maintenance records, 62 patients were categorised as compliant with regular periodontal maintenance visits and 38 as non-compliant. The demographic and clinical characteristics of the study population are presented in (Table 1). No statistically significant differences were observed between the compliant and non-compliant groups in age, gender distribution, smoking status, or history of periodontal disease (p < 0.05), indicating comparability between the groups. The overall implant survival rate in the study population was 93%. Implant survival outcomes in relation to periodontal maintenance compliance are summarised in (Table 2). Among patients compliant with periodontal maintenance, implant survival was observed in 60 of 62 patients (96.8%), whereas implant failure occurred in only 2 patients (3.2%). In contrast, the non-compliant group had a lower implant survival rate: 33 of 38 patients (86.8%) had implant survival and 5 (13.2%) experienced implant failure. The difference in implant survival between the compliant and non-compliant groups was statistically significant (p = 0.03). Correlation analysis revealed a positive association between periodontal maintenance compliance and implant survival, as shown in (Table 3). Spearman's correlation coefficient demonstrated a moderate positive correlation (r = 0.41), which was statistically significant (p < 0.01), indicating that higher compliance with periodontal maintenance was associated with improved implant survival outcomes. Multivariate logistic regression analysis was performed to assess the effect of periodontal maintenance compliance on implant survival, while controlling for potential confounders, including age, gender, smoking status and periodontal history. As shown in (Table 4), periodontal maintenance compliance was a significant predictor of implant survival (Odds Ratio = 3.9; 95% CI: 1.2-12.5; p = 0.02). Other variables did not show a statistically significant association with implant survival.

## Discussion:

The long-term success of dental implants depends not only on surgical and prosthetic precision but also on the maintenance of peri-implant tissue health over time. Periodontal maintenance compliance has increasingly been recognised as a critical factor affecting implant survival, particularly among patients rehabilitated with fixed prosthodontic restorations. The present retrospective cohort study evaluated the correlation between periodontal maintenance compliance and implant survival and demonstrated a significant association between regular maintenance care and improved implant survival outcomes. The results of the present study revealed a higher implant survival rate among patients compliant with periodontal maintenance therapy than among those non-compliant. Patients who adhered to regular maintenance visits exhibited a survival rate of 96.8%, whereas the non-compliant group demonstrated a lower survival rate of 86.8%. This difference was statistically significant, indicating that maintenance compliance plays an important role in the long-term preservation of dental implants. These findings are consistent with previous reports emphasising the importance of supportive periodontal therapy in preventing biological complications around implants [11, 12]. One possible explanation for the higher implant survival observed in compliant patients is the effective control of plaque accumulation and early detection of peri-implant disease during maintenance visits. Regular professional cleaning, reinforcement of oral hygiene instructions and clinical monitoring allow for timely management of peri-implant mucositis before it progresses to peri-implantitis, a condition strongly associated with implant failure [13]. In contrast, non-compliant patients are more likely to experience unchecked plaque accumulation, leading to persistent inflammation and progressive marginal bone loss around implants [14]. The moderate positive correlation observed between periodontal maintenance compliance and implant survival further supports the role of maintenance care in implant longevity. This finding suggests that as compliance with maintenance programs increases, the likelihood of implant survival also improves. Similar observations have been reported by Roccuzzo *et al.* who demonstrated that patients enrolled in regular maintenance programs had significantly lower rates of peri-implant bone loss and implant failure during long-term follow-up [7]. These results reinforce the concept that implant therapy should be viewed as a long-term commitment requiring continuous professional care rather than a one-time intervention. Multivariate logistic regression analysis in the present study identified periodontal maintenance compliance as a significant predictor of implant survival, even after adjustment for potential confounders, including age, gender, smoking status and periodontal history. This finding highlights maintenance compliance as an independent determinant of implant success. Although smoking and a history of periodontitis are well-established risk factors for implant complications, their lack of statistical significance in the present analysis may be attributed to the relatively small number of failures and the controlled clinical environment [15, 16]. The results of this study are particularly relevant for patients with fixed prosthodontic restorations; as such, prostheses may hinder adequate plaque control due to limited access for oral hygiene measures. Fixed restorations can mask early clinical signs of peri-implant inflammation, making regular professional evaluation essential for early diagnosis and intervention [17]. Prosthodontic considerations to optimize implant restoration outcomes-a clinical practice review emphasized that long-term success of implant-supported prostheses depends on proper prosthodontic planning, maintenance protocols and effective plaque control around implants. The authors highlighted that regular follow-up care and patient compliance with maintenance therapy significantly contribute to peri-implant tissue stability and restoration longevity. Factors affecting the success of dental implant treatments reported that implant survival is strongly influenced by patient-related factors such as oral hygiene practices, periodontal health and adherence to supportive maintenance programs. The study further suggested that inadequate maintenance compliance increases the risk of peri-implant inflammation, marginal bone loss and implant failure in fixed prosthodontic restorations [18, 19]. Therefore, the clinician's role in educating patients about the importance of maintenance compliance becomes crucial for ensuring long-term treatment success. Despite its valuable findings, the present study has certain limitations. The retrospective design relies on the accuracy and completeness of patient records, which may introduce selection or information bias. Additionally, implant survival was used as the primary outcome measure and parameters such as marginal bone loss and peri-implant soft tissue indices were not evaluated. Future prospective longitudinal studies incorporating comprehensive clinical and radiographic assessments are recommended further to elucidate the impact of maintenance compliance on peri-implant health. Within these limitations, the present study underscores the importance of periodontal maintenance compliance as a key factor influencing implant survival in patients with fixed prosthodontic restorations. Emphasising regular maintenance visits and patient education may significantly enhance long-term implant outcomes and reduce the incidence of implant-related complications.

## Conclusion:

Within the limitations of the present study, it can be concluded that periodontal maintenance compliance plays a significant role in the long-term survival of dental implants in patients rehabilitated with fixed prosthodontic restorations. Patients who adhered to regular periodontal maintenance visits demonstrated higher implant survival rates compared to those who were non-compliant. These findings highlight the importance of supportive periodontal therapy and continuous professional monitoring in maintaining peri-implant tissue health and preventing biological complications. Therefore, patient education and motivation toward regular maintenance care should be considered an integral component of implant therapy to enhance long-term clinical outcomes.

## Advancement to knowledge:

This study provides insight into the impact of periodontal maintenance compliance on the long-term survival of dental implants following fixed prosthodontic restorations. It emphasizes the importance of regular supportive periodontal care in improving implant prognosis, reducing peri-implant complications and enhancing the longevity of prosthodontic treatment outcomes.

## Figures and Tables

**Table 1:** Demographic and clinical characteristics of the study population

**Variable**	**Compliant (n = 62)**	**Non-compliant (n = 38)**	**p value**
Mean age (years)	46.3 ± 9.1	47.8 ± 8.7	0.42
Gender (Male/Female)	34 / 28	21 / 17	0.95
Smokers	12 (19.4%)	9 (23.7%)	0.61
History of periodontitis	18 (29.0%)	13 (34.2%)	0.58

**Table 2:** Association between periodontal maintenance compliance and implant survival

**Maintenance compliance**	**Implant survived**	**Implant failed**	**Total**	**p value**
Compliant	60 (96.8%)	2 (3.2%)	62	
Non-compliant	33 (86.8%)	5 (13.2%)	38	0.03*
* Statistically significant
(p < 0.05), Chi-square test

**Table 3:** Correlation between periodontal maintenance compliance and implant survival

**Variable**	**Correlation coefficient (r)**	**p value**
Maintenance compliance vs implant survival	0.41	<0.01*
*Statistically significant, Spearman's correlation

**Table 4:** Multivariate logistic regression analysis for predictors of implant survival

**Variable**	**Odds Ratio (OR)**	**95% Confidence Interval**	**p value**
Maintenance compliance	3.9	1.2-12.5	0.02*
Age	1.1	0.9 - 1.3	0.27
Gender	1	0.4-2.4	0.94
Smoking	0.7	0.2-2.3	0.56
Periodontal history	0.8	0.3-2.5	0.71
*Statistically significant (p < 0.05)

## References

[1] Albrektsson T, Donos N (2012). Clin Oral Implants Res..

[2] Esposito M (1998). Eur J Oral Sci..

[3] Berglundh T, Lindhe J (1996). J Clin Periodontol..

[4] Serino G, Ström C (2009). Clin Oral Implants Res..

[5] Lindhe J (2008). J Clin Periodontol..

[6] Axelsson P, Lindhe J (1981). J Clin Periodontol..

[7] Roccuzzo M (2010). Clin Oral Implants Res..

[8] Wilson TG-Jr (1996). Periodontol 2000..

[9] Cardaropoli D (2012). J Periodontol..

[10] Heitz-Mayfield LJA (2008). J Clin Periodontol..

[11] Heitz-Mayfield LJA (2005). J Clin Periodontol..

[12] Roos-Jansåker AM (2007). Swed Dent J Suppl..

[13] Zitzmann NU, Berglundh T (2008). J Clin Periodontol..

[14] Ko KW (2024). J Dent Sci..

[15] Bain CA, Moy PK (1993). Int J Oral Maxillofac Implants..

[16] Karoussis IK (2003). Clin Oral Implants Res..

[17] Wilson TG-Jr (2009). J Periodontol..

[18] Nogueron-Dorca N (2026). Front Oral Maxillofac Med..

[19] https://izlik.org/JA63NK49BM.

